# Perceived Healthcare Discrimination and Its Impact on Mistrust and Delayed Care Among Sexual and Gender Minority Young Adults in Spain

**DOI:** 10.1007/s10508-026-03516-z

**Published:** 2026-09-03

**Authors:** Andrea Miranda-Tena, Francisco J. Sanmartín, Judith Velasco

**Affiliations:** 1 Department of Psychology, University of Córdoba, Córdoba, 14071 Spain; 2 Department of Research and Psychology in Education, Complutense University of Madrid, Madrid, Spain; 3 Maimonides Institute for Biomedical Research of Cordoba, Córdoba, Spain; 4 Reina Sofia University Hospital, Córdoba, Spain

**Keywords:** LGBTIQ +, Sexual and gender minorities, Discrimination, Gender dysphoria, Sexual orientation, Healthcare

## Abstract

Sexual and gender minority (SGM) individuals face discrimination in health care, compromising both well-being and access to services. Such experiences may foster expectations of mistreatment and mistrust of health care professionals, contributing to delays in care with potentially adverse consequences. This study examined perceived discrimination, mental and physical health, trust in providers, and care-seeking behavior among young adults in Spain, comparing SGM participants with their cisgender heterosexual peers. A cross-sectional online survey was conducted with 217 participants (*M* = 21.5 and *SD* = 4.6), of whom 144 identified as cisgender heterosexual and 73 as SGM. Qualitative open-text responses were analyzed using reflexive thematic analysis. Data were collected between January and April 2025. SGM participants reported higher levels of perceived discrimination, greater psychological distress, lower trust in providers, and more delays in seeking care than cisgender heterosexual participants. Although perceived discrimination was associated with psychological distress, trust, and delayed care at the bivariate level, discrimination did not emerge as an independent predictor of health outcomes in multivariate regression models. Qualitative findings illustrated that discrimination (including misgendering, medical paternalism, and moral judgement) was experienced in everyday clinical encounters and linked to identity concealment and erosion of trust. These findings suggest that universal health coverage and legal protections are insufficient to ensure equitable care in the absence of affirming, culturally competent care. Structural reforms, inclusive protocols, and provider training are essential to reduce health disparities affecting SGM populations.

## Introduction

Primary health care systems are designed to ensure broad accessibility and continuity of care, contributing to improved population health outcomes. In health care contexts, discrimination refers to differential or unfair treatment based on personal characteristics such as sexual orientation or gender identity, which may manifest through overt exclusion, stigmatizing interactions, or institutional practices that limit access to appropriate care (Skuban-Eiseler et al., 2024). Global evidence continues to highlight persistent challenges, including access barriers such as long waiting times and inefficient patient flow (Sokhela et al., 2013), cultural and communication difficulties (Peprah et al., 2024), compromised care quality (Davey et al., 2013), and broader systemic issues (Mchunu et al., 2022). These challenges are especially pronounced among sexual and gender minority (SGM) populations, who frequently report discriminatory experiences ranging from subtle exclusion to overt denial of care (Arnold & Dhingra, 2020). Such experiences have been linked to poorer mental health outcomes, compromised interactions with health care providers, and delays or avoidance of care (Ayhan et al., 2020; Yu et al., 2023). Importantly, these disparities are not confined to settings with limited legal protections or under-resourced health systems. They persist even in countries with universal coverage and progressive legal frameworks. Spain, for example, is internationally recognised for its inclusive LGBTIQ+ legislation and publicly funded health system; yet, evidence indicates that SGM individuals continue to face discriminatory treatment in primary care. A survey conducted by the Spanish Federation of Lesbians, Gays, Trans, Bisexuals, and Intersex People (Federación Estatal de Lesbianas, Gais, Trans y Bisexuales, 2019) found that 21.7% of transgender respondents accessed primary care only occasionally and 5.8% avoided it altogether, citing prior discriminatory experiences such as misgendering, “deadnaming,” and moral judgements. Recent research further illustrates how discrimination is experienced in health care settings by SGM populations. Studies with non-binary individuals in Catalonia (Gómez-Ibáñez et al., 2024), transgender inmates in Barcelona (Sererols-Serra & Leyva-Moral, 2025), and LGBTIQ+ refugees (Leyva-Moral et al., 2026) document persistent barriers related to identity recognition, stigma, and institutional exclusion, and highlight a continuing gap between formal legal equality and the provision of inclusive care (Villemure et al., 2023).

Building on this evidence, a growing body of research has sought to explain the mechanisms by which health care discrimination impacts well-being and care-seeking behavior. Several theoretical frameworks provide insight into these dynamics. Minority stress theory (Meyer, 2003), for example, conceptualizes discrimination-related stressors as cumulative processes operating through both external events (e.g., unequal treatment) and internal mechanisms (e.g., anticipation of rejection), ultimately leading to adverse mental and physical outcomes over time. In Spain, a national study conducted by the Carlos III Health Institute (Belza et al., 2024) reported that 80% of transgender and non-binary individuals experienced fear or discrimination when accessing primary care, a finding echoed by documented gaps in provider knowledge of LGBTIQ+ health needs (Santander-Morillas et al., 2022). Complementing this perspective, the psychological mediation model (Hatzenbuehler, 2009) highlights cognitive and affective processes such as vigilance, rumination, and avoidance as key mediators linking stigma to health outcomes. These processes may shape how SGM individuals anticipate and navigate clinical encounters, thereby contributing to delayed or foregone care. Finally, the health equity promotion model (Fredriksen-Goldsen et al., 2014) situates individual experiences within broader structural contexts, emphasising how institutionalised discrimination can erode trust, continuity of care, and long-term engagement with health services.

Together, these frameworks support the notion that discrimination, even when subtle or diffuse, can shape patterns of disengagement and mistrust. However, there remains a need for empirical studies that jointly examine perceived discrimination, health outcomes, trust in providers, and care-seeking behaviors, particularly in contexts such as Spain, where progressive legal protections coexist with persistent experiences of stigma. The present study addresses these gaps through a mixed methods approach. Quantitatively, it compares SGM and cisgender heterosexual young adults across five domains: perceived discrimination, psychological distress, somatic symptoms, trust in providers, and delayed care. Qualitatively, it examines participants’ lived experiences of discrimination in primary care to contextualise how bias is experienced in routine clinical interactions. Accordingly, this study investigates the following research questions:

1. Do SGM young adults in Spain report higher levels of personal and systemic discrimination in primary care compared with cisgender heterosexual peers?
  i. **Hypothesis 1 (H1):**
    SGM young adults will report higher levels of both personal and systemic discrimination.
2. Are higher levels of perceived discrimination associated with poorer psychological and physical health, lower trust in providers, and greater delays in care?
  i. **Hypothesis 2 (H2):**
    Higher perceived discrimination will be associated with poorer psychological health, poorer physical health, lower trust in providers, and greater delays in care-seeking.
3. How do SGM participants describe their experiences of discrimination in primary care, and in what ways do these encounters manifest in clinical interactions?
  i. **Hypothesis 3 (H3):**
    SGM participants will describe experiences of discrimination that manifest through interpersonal and systemic mechanisms within primary care.

This study used a cross-sectional, quantitative dominant mixed methods design with a supplementary qualitative component (Johnson & Christensen, 2014). Specifically, a convergent approach was used, whereby quantitative and qualitative data were collected concurrently and integrated at the interpretation stage. Consistent with this type of mixed research, the qualitative component was not intended to constitute a standalone qualitative analysis, but to provide illustrative context to the quantitative findings. The quantitative component assessed perceived discrimination, psychological distress, somatic symptoms, trust in health care providers, and delays in care among SGM and cisgender heterosexual participants. The qualitative component examined participants’ subjective experiences of discrimination in primary care through thematic analysis (TA) of open-text responses. A single open-ended question was included at the end of the survey, inviting participants to voluntarily describe any discrimination experiences.

## Method

### Participants

Participants were recruited using a non-probabilistic convenience sampling strategy. Recruitment was conducted online through social media platforms, mailing lists, and LGBTIQ+ community organisations based in Spain. For the SGM subgroup, advertisements invited adults of diverse sexual orientations and gender identities to participate in a study examining experiences in primary health care.

Inclusion criteria were: (1) being between 18 and 40 years of age; (2) residing in Spain, and (3) providing informed consent. Participants were excluded if they did not complete key demographic variables required for group classification, including sexual orientation or gender identity.

### Measures

#### Demographics and Health Care Utilization

Participants reported age, gender identity, sexual orientation, racial/ethnic identity, educational attainment, chronic health conditions, and region of residence. Health care utilization variables included frequency of primary care visits, reasons for consultation, type of provider consulted, continuity of care, and subjective evaluations of visit quality.

#### General Discrimination

General perceptions of discrimination in health care were assessed using an adapted version of the General Discrimination Scale in Health care Settings (LaVeist et al., 2000), originally designed to assess perceived racism. For the present study, the items were adapted and translated into Spanish to reflect the experiences of LGBTIQ + populations. The scale includes four items rated on a 5-point Likert scale ranging from 1 (*strongly disagr*ee) to 5 (*strongly agree*). All items were reverse coded to produce total scores ranging from 4 to 20, with higher scores indicating lower perceived discrimination. Internal consistency was acceptable (*α* = .76).

#### Perceived Discrimination

Discrimination by primary care providers was measured using the Discrimination in Medical Settings Scale (Peek et al., 2011), adapted from the Everyday Discrimination Scale (Williams et al., 1997) and translated into Spanish. The scale includes seven items assessing perceived disrespect, dismissiveness, or unfair treatment during health care encounters, rated on a 5-point Likert scale (1 = *never* to 5 = *always*). Total scores ranged from 7 to 35, with higher scores indicating greater perceived discrimination. Internal consistency was high (*α* = .89).

#### Personal Discrimination in Health Care

Personal experiences of discrimination were assessed using a single item adapted from the Experiences of Discrimination Scale (Krieger et al., 2012): “When receiving care in primary health care, have you ever been discriminated against, prevented from doing something, hassled, or made to feel inferior because of your sexual orientation or gender identity?” Responses were provided on a 4-point scale ranging from 1 (*never*) to 4 (*four or more times*), with higher scores indicating greater perceived discrimination. The instrument was administered in Spanish using a forward–backward translation. Reliability for this item was acceptable (α = .74).

#### Psychological Distress

Psychological distress was measured using the 12-item General Health Questionnaire (GHQ-12; Goldberg, 2011). Items were rated on a 4-point Likert scale (0 = *not at all* to 2 = *much more than usual*), with higher scores indicating greater distress. A total score of 3 or higher is indicative of possible mental health difficulties. The validated Spanish version (Sánchez-López & Dresch, 2008) was used (*α* = .76).

#### Somatic Symptoms

Somatic symptoms were assessed using the Patient Health Questionnaire-15 (Kroenke et al., 2002), which measures 15 symptoms experienced during the previous 4 weeks. Items were rated on a 3-point Likert scale (0 = *not bothered at all* to 3 = *bothered a lot*), yielding total scores from 0 to 30. Symptom severity is classified as low (5–9) moderate (10–14), or high (> 15). The validated Spanish adaptation was used (Ros Montalbán et al., 2010), with adequate internal consistency of .78 in the current sample.

#### Trust in Providers

Trust in health care professionals was measured using a Spanish-translated version of the Trust in Physician Scale (Anderson & Dedrick, 1990), an 11-item instrument assessing perceptions of physicians’ competence, reliability, and respect towards the patient. Items are rated on a 5-point Likert scale (1 = *strongly disagree* to 5 = *strongly agree*), with higher scores indicating greater trust. Internal consistency in the present sample was high (*α* = .85).

#### Delayed Care

Delayed care was assessed using three dichotomous items adapted from prior research (Kannan & Veazie, 2014; Mollborn et al., 2005) and translated into Spanish: (a) Some people avoid visiting their doctor even when they suspect they should. Would you say this is true for you, or not true?, (b) In the past 12 months, was there any time when you did not seek medical care that you believed you needed; and (c) In the past 12 months, was there any time when you delayed seeking medical care that you believed you needed? Affirmative responses were coded to indicate a greater tendency to delay care. Internal consistency in the present sample was acceptable (*α* = .70).

### Procedure

Data were collected anonymously using a secure online survey platform. Recruitment materials included a brief description of the study aim and a link to the survey. Upon accessing the survey, participants were presented with an informed consent form detailing the study objectives, the voluntary nature of participation, confidentiality and data protection measures, and the option to withdraw at any time without consequences. Only participants who provided informed consent were able to proceed to the questionnaire.

The survey included demographic questions, measures of discrimination, health and care-related outcomes, and a single open-ended question inviting participants to describe experiences of discrimination in primary care. All responses were recorded anonymously, and no personally identifiable information was collected. No financial or other incentives were offered for participation.

### Data Analysis

Normality was assessed using Shapiro–Wilk tests, indicating that only the GHQ-12 and Trust in Physicians scales met the assumption of normality. Homogeneity of variances was confirmed for all variables except personal discrimination (PD-U). Descriptive statistics and Pearson's correlations were calculated for all study variables, and internal consistency was assessed using Cronbach’s alpha. Group differences between SGM and cisgender heterosexual participants were examined using independent-samples *t-tests* or Mann–Whitney *U* tests, as appropriate, while Chi-square tests were applied to categorical variables. Effect sizes were reported as Cohen’s *d* for continuous variables and φ for categorical variables.

To examine the predictive role of perceived discrimination, multiple linear regression models were conducted with psychological distress (GHQ-12) and somatic symptoms (PHQ-15) as dependent variables, and the three discrimination measures (PD-U, PD-M, and GD) entered as predictors. The three discrimination measures were entered simultaneously to examine their relative contributions while accounting for overlapping but conceptually distinct dimensions of discrimination, including general perceptions, interpersonal treatment in medical settings, and personal experiences of discrimination. No missing data were present. Regression assumptions were verified and met, and variance inflation factors (VIFs) were below 2.0, indicating no multicollinearity. Sample size adequacy for the planned regression models was calculated using the PAMLj module in jamovi. Assuming a moderate effect size (*f*^2^ = .25; equivalent to partial *η*^2^ = .20), *α* = .05, 90% power, and three predictors, the minimum required sample size was *N* = 61. With the achieved sample size (*N* = 217), power analyses indicated sufficient sensitivity to detect small effects (*f*^2^ ≈ .05). For the SGM group (*n* = 73), the minimum detectable effect size was moderate (*f*^2^ = .206). Statistical significance was set at *p* < .05. All analyses were conducted using JAMOVI version 2.3 (The jamovi project, 2023).

Qualitative data, consisting of open-ended responses describing discrimination experiences in primary care, were analyzed using reflexive thematic analysis with an inductive orientation. Given the exploratory and supplementary nature of the qualitative component and the use of brief open-text responses, this approach was selected for its flexibility and suitability for identifying patterned meanings. Analyses followed an inductive, iterative process aligned with the six phases outlined by Braun and Clarke (2006). During familiarisation, two researchers read all responses multiple times to become immersed in the data and note initial impressions. In the initial coding phase, responses were coded inductively at a semantic level to capture explicit content related to discrimination experiences. Codes were then examined for shared patterns of meaning and organised into provisional themes, which were iteratively reviewed and refined through discussion to ensure internal coherence and clear distinction between themes. Final themes were subsequently defined and named.

Consistent with the reflexive thematic analysis approach, themes were treated as analytic constructions rather than as entities assumed to emerge directly from the data. Given the limited number of responses and the illustrative role of the qualitative component, thematic saturation was not treated as an analytic goal. Instead, the analysis focused on achieving thematic coherence, in line with contemporary methodological recommendations (Braun & Clarke, 2019, 2021). Atlas.ti software was used for data management and coding. To increase trustworthiness, two researchers (J.V. and F.J.S.) independently coded a subset of responses and resolved differences through discussion.

## Results

### Participant Characteristics

The initial sample consisted of 228 participants. After applying exclusion criteria (i.e., age over 40 years, missing data regarding gender identity or sexual orientation), the final sample comprised 217 individuals aged 18–40 years (*M* = 21.5, *SD* = 4.6).

Of these, 144 (66.4%) identified as cisgender heterosexual, including 32 men (22.2%) and 112 women (77.8%). The remaining 73 participants (33.6%) were classified as SGM, identifying as gay or lesbian (32.9%), bisexual (60.3%), or another sexual orientation (5.5%). Within the SGM group, 61.6% identified as cisgender women, 27.4% as cisgender men, and 11.0% as transgender or non-binary.

Most participants (94.0%) identified as White. Educational attainment was high, with the majority reporting completion of some university-level education. Most participants reported no chronic health conditions. Group-specific descriptive statistics are presented in Table 1.

**Table 1:** Demographic characteristics of the sample separated by group

Variable		Cis-straight (*n* = 144)	SGM (*n* = 73)	Comparisons	
		*M (SD)*	*M (SD)*	*U*	*p*
Age		20.6 (3.7)	23.2 (5.6)	3592	< .001
		*n* (%)	*n* (%)	*χ*^*2*^	*p*
Sex	Male	32 (22.2)	24 (32.8)	2.34	.126
	Female	112 (77.8)	49 (67.2)		
Gender identity	Cisgender men	32 (22.2)	20 (27.4)	18.1	.001
	Cisgender women	112 (77.8)	45 (61.6)		
	Transgender men	–	3 (4.1)		
	Transgender women	–	1 (1.4)		
	Nonbinary	–	4 (5.5)		
Sexual Orientation	Heterosexual	144 (100)	1 (1.4)	213	< .001
	Gay/Lesbian	–	24 (32.9)		
	Bisexual	–	44 (60.2)		
	Other/Prefer not to label	–	4 (5.5)		
Highest level of education	High school	1 (0.7)	–	17.7	.003
	USE	5 (3.5)	7 (9.6)		
	VET	4 (2.8)	8 (10.9)		
	College	120 (83.3)	43 (58.9)		
	Master	13 (9.0)	14 (19.2)		
	Doctorate	1 (0.7)	1 (1.4)		
Employment status	Student	135 (93.8)	58 (79.5)	26.6	< .001
	Employed	22 (15.3)	17 (23.3)		
	Unemployed	–	4 (5.5)		
	Retired	–	1 (1.4)		
Race/ethnicity	White	139 (96.5)	66 (90.4)	3.84	.428
	Black	1 (0.7)	1 (1.4)		
	Asian	1 (0.7)	1 (1.4)		
	Latinx	2 (1.4)	4 (5.4)		
	Romani	1 (0.7)	1 (1.4)		

*Note*. USE = Upper Secondary Education; VET = Vocational Education and Training

*Note.* Percentages in the employment status category may exceed 100% due to some participants responding various categories

### Primary Care Utilization

Participants reported varying patterns of primary care utilization. Approximately one-third (31.8%, *n* = 69) reported visiting a primary care provider once every 6 months, while 25.3% (*n* = 55) reported visits approximately once every 3months. Preventive check-ups were the most frequently cited reason for seeking care (68.7%, *n* = 149).

Most participants (87.1%, *n* = 189) reported being primarily attended by a physician, and two-thirds (66.8%, *n* = 145) indicated continuity of care, typically consulting the same provider across visits. Group-specific utilization patterns are presented in Table 2.

**Table 2:** Primary care utilization patterns and characteristics of visits

Variable	Total sample (*N* = 217)	Cis-straight (*n* = 144)	SGM (*n* = 73)	Comparisons		
	*n* (%)	*n* (%)	*n* (%)	*χ*^2^	*p*	
Use of PCS	> 1 time/month	5 (2.3)	3 (2.1)	2 (2.7)		
	1 time/month	17 (7.8)	10 (6.9)	7 (9.6)		
	> 1 time/3 months	55 (25.3)	31 (21.5)	24 (32.9)		
	> 1 time/ 6 months	69 (31.8)	45 (31.3)	24 (32.9)	7.36	.289
	1 time/year	54 (24.9)	42 (29.2)	12 (16.4)		
	1 time/ 2 years	6 (2.8)	4 (2.8)	2 (2.7)		
	None	11 (5.1)	9 (6.3)	2 (2.7)		
Reason for consultation	Preventive care	149 (68.7)	101 (70.1)	48 (65.8)		
	Acute illness	50 (23.0)	35 (24.3)	15 (20.5)	4.31	.116
	Chronic condition	18 (8.3)	8 (5.6)	10 (13.7)		
Primary provider	Physician	189 (87.1)	125 (86.8)	64 (87.7)		
	Nurse	8 (3.7)	6 (4.2)	2 (2.7)	0.29	.866
	Both	20 (9.2)	13 (9.0)	7 (9.6)		
Same professional	Yes	145 (66.8)	98 (68.1)	47 (64.4)	0.15	.696
	No	72 (33.2)	46 (31.9)	26 (35.6)		
Evaluation of the visit	1/5	7 (3.2)	5 (3.5)	2 (2.7)		
	2/5	26 (12.0)	17 (11.8)	9 (12.3)		
	3/5	74 (34.1)	46 (31.9)	28 (38.4)	1.07	.899
	4/5	82 (37.8)	57 (39.6)	25 (34.2)		
	5/5	28 (12.9)	19 (13.2)	9 (12.3)		

*Note.* SGM = Sexual and Gender Minorities; PCS = Primary Care Services;

### Group Differences in Discrimination

On the single-item measure of personal discrimination in health care (PD-U), SGM participants reported significantly higher levels of discrimination than cisgender heterosexual participants, *χ*^2^(3, *N* = 217) = 24.7, *p* < .001, *γ* = .76. No significant group differences were observed on the multi-item discrimination scale (PD-M), *U* = 4587, *p* = .123, *d* = .127. In contrast, scores on the General Discrimination Scale differed significantly between groups, *t* = 4.71, *p* < .001, *d* = .68, with SGM participants reporting lower scores (*M* = 12.2, *SD* = 3.5) than cisgender cisheterosexual participants (*M* = 14.6, *SD* = 3.6), indicating greater perceived discrimination.

### Group Differences in Health and Behavioral Outcomes

SGM participants reported significantly higher psychological distress, as measured by the GHQ-12, than cisgender heterosexual participants, *t* = − 2.71, *p* = .007, *d* = .37. They also reported significantly lower trust in health care providers, *t* = 2.21, *p* = .028, *d* = .32, and were more likely to report delays in seeking care, *U* = 4247, *p* = .017. No significant group differences were observed for physical health complaints, *U* = 4497, *p* = .082 (Table 3).

**Table 3:** Group differences in psychological and health care indicators

Variable	Cis-straight (*n *= 144)	SGM (*n* = 73)
	*M* (*SD*)	*M* (*SD*)
GHQ-12	16.8 (6.5)	19.4 (7.4)
Trust in professionals	37.4 (7.9)	34.9 (7.5)
	Mdn (IQR)	Mdn (IQR)
PHQ-15	9.0 (8.3)	11.0 (6.0)
Delayed care	1.0 (3.0)	2.00 (2.0)

### Bivariate Correlations

Bivariate analyses indicated that perceived discrimination was associated with poorer mental health and care-related outcomes. Personal discrimination was significantly associated with psychological distress only when measured using the multi-item scale (PD-M). Both PD-U and PD-M were significantly correlated with lower trust in health care providers and greater delays in care. In addition, general discrimination (GD) was negatively correlated with trust in providers (Table 4).

**Table 4:** Bivariate correlations among mental health and health care utilization variables

Variables	α	1	2	3	4	5	6	7
1. PD-U	–	–	.58***	− .42***	.17	.05	.38**	.24*
2. PD-M	.89		–	− .27*	.24*	.15	− .36**	.28*
3. GD	.86			–	− .12	.06	.33*	− .22
4. GHQ-12	.91				–	.60***	− .20	.18
5. PHQ-15	.80					–	–.27*	.18
6. Trust in professionals	.85						–	–.17
7. Delayed care	.73							–

*Note.* PD-U = Perceived Discrimination – Single Item; PD-M = Perceived Discrimination – Multiple Items (PD-M); GD = General Discrimination; GHQ-12 = General Health Questionnaire; PHQ-15 = Patient Health Questionnaire

*p < .05 **p < .01 *** *p* < .001

### Regression Analyses

Multiple regression analyses were conducted to examine whether the three discrimination indices (PD-U, PD-M, and GD) predicted psychological distress (GHQ-12), physical health symptoms (PHQ-15), trust in health care providers, and delays in care. None of the models reached overall statistical significance. Although several bivariate correlations were observed, none of the discrimination measures emerged as a statistically significant independent predictor when all three indices were entered simultaneously into the models.

A total of 23 open-ended responses were collected, 60.8% of which were provided by SGM participants. This item was not intended to support an in-depth qualitative analysis, but to complement the quantitative findings related to the study’s first objective of examining experiences of discrimination in primary care. Given the limited number of open-ended responses, the qualitative findings should be understood as illustrative accounts that provide contextual insight into participants’ experiences, not as representative of the broader SGM population. The primary themes identified are summarised below.

### Lack of Information

This theme captured participants’ comments of insufficient knowledge among health care professionals regarding issues specific to SGM populations. Participants described gaps in providers’ familiarity with SGM-related health needs, which were experienced as barriers to appropriate care.

Two subthemes were identified. First, limited knowledge of STI prevention protocols and eligibility criteria. As one participant noted, “I didn’t feel they had sufficient knowledge about STIs and prevention protocols […].” Second, a lack of awareness of diverse gender identities, including transgender and non-binary identities (“Denial of my reality, complete, or partial ignorance of what it means to be a trans person.”).

### Diagnostic Dismissal and Delays

This theme described participants’ experiences of having symptoms minimised, misattributed, or insufficiently investigated by health care professionals. These experiences illustrate difficulties in obtaining accurate diagnoses and timely referrals, particularly when sexual orientation or gender identity was not initially disclosed. One participant described a prolonged period of misdiagnosis before relevant sexual history was acknowledged:

[...] I lived with Mycoplasma Genitalium for more than 2 years, treated as if it were a urinary infection. One day I casually mentioned that my ex-partner was a man, and the treatment changed completely—they did a full STI screening and finally detected the mycoplasma. It took a long time and several treatments to clear the infection. The symptoms are suspected to have become chronic, with prostate involvement, but I was never referred to infectious disease specialists.

### Medical Paternalism

This theme described experiences in which health care professionals asserted authority over medical decisions while discounting patients’ perspectives. Participants characterised these interactions as patronising, directive, or dismissive of their expressed needs. As one participant stated, “They told me what to do in my private life as if they were my mother and were completely unaware of the care I actually needed. I had to argue and prove they were wrong.”

### Interactions During Consultations

This theme reflected experiences of unprofessional or dismissive communication during consultations. Participants described a lack of empathy and the use of condescending language, which contributed to negative perceptions of the clinical encounter. One participant noted, “The doctors were condescending in the way they spoke to me.” This illustrates how communication style, rather than clinical content alone, can shape experiences of care.

### Gender Identity Recognition

This theme captured instances of misgendering, including the use of incorrect pronouns or “deadnames,” often based on assumptions related to sex assigned at birth. As one participant described, “Systematic use of a name that is not mine, systematic use of feminine pronouns, which is incorrect […].”

### Concealment

This theme reflected participants’ deliberate decisions to withhold information about their sexual orientation or gender identity in medical settings. Concealment was described as a protective strategy motivated by anticipated stigma or discomfort. As one participant explained, “I don’t seem ‘stereotypically gay’ and never discuss these things with my doctor, so I’d be surprised if they knew about my sexual orientation.”

### Moral Judgements

This theme described experiences of moralizing remarks related to sexual behavior, sexual orientation, or gender identity. Participants perceived these comments as judgmental and stigmatizing. One participant recounted:

I experienced two discriminatory situations recently. One was when I received my HIV diagnosis and was told things like “You should have been more careful” or “if you didn’t use protection before, you’ll probably do it again.” The other was when they evaluated whether to give me the monkeypox vaccine and asked, “how are you handling your promiscuity?”

### Denial of Treatment

This theme reflected experiences in which participants perceived that access to preventive interventions (e.g., vaccines and PrEP) was withheld based on providers’ assumptions about sexual behavior or risk, illustrating perceived barriers to preventive care. One participant explained:

I requested a vaccine covered by public health that protects against some STIs, and my doctor didn’t think I needed it because I had a partner. I explained we were in an open relationship, and that despite taking precautions, I would feel safer with the vaccine. [...] I remain unvaccinated’)

### Weight-Based Discrimination

This theme described experiences of stigma related to body weight. It reflects repeated instances in which health concerns are attributed to weight, delaying accurate diagnosis and referral. The participant recounted:

In my case, I’ve experienced more fatphobia than any other type of discrimination. Just three years ago, with a new doctor, I was finally diagnosed with a skin condition that had always been blamed on my weight. They never investigated further or referred me to specialists

## Discussion

Extensive research has documented high levels of discrimination among sexual and gender minorities (Stacey et al., 2022; Valdiserri et al., 2019), which are consistently associated with poorer overall well-being (Stacey & Wislar, 2023). Although medical settings are formally regulated by anti-discrimination policies, heteronormative assumptions often persist in clinical practice, contributing to ongoing disparities in care for SGM individuals that may adversely impact their health (Mansh et al., 2015). In this context, the present study examined perceived discrimination in primary care among young adults in Spain and its association with health outcomes and care-seeking behaviors using a mixed methods approach. Given Spain's distinctive context (as a leading country in the advancement of LGBTIQ+ rights and the provision of universal, publicly funded health care), this study offers insights that can enrich the existing literature on SGM experiences within medical settings.

Regarding the first research question, quantitative results did not reveal significant group differences in reported experiences of personal discrimination in primary care. However, differences emerged on the general discrimination scale, suggesting greater awareness of systemic discrimination among SGM participants compared with their cisgender heterosexual peers. This pattern diverges from previous research documenting higher levels of direct discrimination among SGM populations (Ayhan et al., 2020; Müller, 2016). Possible explanations include the relatively young age of participants, their limited engagement with primary care services, or increased provider sensitivity within the studied context.

Qualitative findings add important nuance to these results by illustrating how discrimination may be enacted through both overt and subtle mechanisms, including misgendering, symptom dismissal, moralizing comments, paternalistic interactions, and denial of care. These experiences appeared to undermine participants’ sense of safety, reinforced identity concealment, and contribute to care avoidance, particularly among those acutely aware of systemic inequities affecting SGM populations. Such patterns are consistent with psychological mediation processes described in Minority Stress Theory, whereby anticipated or internalised stigma shapes health-related behaviors.

Interestingly, cisgender heterosexual participants also reported negative experiences in medical settings, particularly related to weight-related stigma and moral judgement. Although these experiences differ in content from those reported by SGM participants, their presence may partially obscure group differences in quantitative analyses. Importantly, the key distinctions lay not in the presence of negative encounters, but in how these experiences were framed and interpreted. Whereas cisgender heterosexual participants tended to describe such interactions as isolated or related to professional conduct, SGM participants were more likely to situate similar experiences within a broader context of systemic discrimination. This distinction is consistent with previous research indicating that stigmatizing communication, including weight-based judgement, has been shown to be common in primary care across patient groups, albeit shaped by different social meanings and attributional processes (Puhl & Heuer, 2009).

As hypothesised, higher levels of perceived discrimination were associated with poorer mental health, lower trust in health care providers, and greater delays in seeking care. Given the cross-sectional design of the study, these associations cannot be interpreted as causal. Rather, they reflect associative patterns that may be shaped by reciprocal or cumulative processes over time. Consistent with prior research, diminished trust appears not merely as an attitudinal outcome, but as a key factor associated with disclosure, adherence, and sustained engagement with health care (Zha et al., 2023). Erosion of trust during early adulthood is particularly concerning, as it may establish long-term patterns of disengagement from preventive care, compounded by the disproportionate burden of chronic illness reported among SGM populations (Dawson et al., 2023; Franklin, 2023).

Nonetheless, when entered into regression models, discrimination measures did not emerge as statistically significant independent predictors of health outcomes or behavioral indicators. One possible explanation is that the effects of discrimination do not operate in isolation but accumulate over time, consistent with minority stress theory and the health equity promotion model (Lick et al., 2013). From this perspective, discrimination may function less as a discrete, measurable predictor and more as a pervasive background stressor interacting with both structural and individual-level variables. This conceptualisation helps explain why robust bivariate associations did not translate into statistically significant effects in the multivariate models. When considered simultaneously, overlapping dimensions of discrimination, combined with limited statistical power in the SGM subgroup, may have attenuated independent effects. Importantly, these null findings should not be interpreted as evidence that discrimination lacks predictive relevance, but rather as reflecting certain methodological limitations of the present study. Future research should employ longitudinal and intersectional methodologies to better capture these cumulative processes and to identify conditions under which discrimination manifests in tangible health deterioration.

Finally, Spain’s sociopolitical context further underscores these findings. Despite universal health care coverage and progressive legal protections, inclusive and affirming care is not guaranteed in everyday clinical practice. Achieving equity requires culturally competent approaches, institutional accountability, and patient-centred care that actively counteract stigma. These results align with evidence from other European settings (Villemure et al., 2023) and contribute to a growing body of research demonstrating how health care discrimination undermines trust and perpetuates health disparities among SGM populations (Ayhan et al., 2020; Fredriksen-Goldsen et al., 2014; Hatzenbuehler, 2009).

### Policy Implications

Addressing health care disparities affecting SGM populations requires coordinated action across multiple levels of the health system. At the provider level, cultural competence training has demonstrated benefits for improving knowledge, attitudes, and clinical practices towards LGBTIQ + patients (Yu et al., 2023). Integrating SGM health content into undergraduate medical education and continuing professional development, particularly around inclusive communication and SGM-specific health needs, aligns with established curricular recommendations (Solotke et al., 2019). Existing initiatives, such as Harvard Medical School’s Sexual and Gender Minority Health Equity Initiative (Keuroghlian et al., 2022), illustrate the potential of structured curricular reforms to reduce bias and foster patient trust (Pedersen & Corcoran, 2021).

At an institutional level, accountability mechanisms including anonymous reporting systems, equity audits, and systematic patient feedback processes are essential for identifying and addressing discrimination in care delivery. In addition, clinical environments should visibly signal inclusivity through gender-neutral language, inclusive intake forms, and affirming signage (Practice Committee of the American Society for Reproductive Medicine, 2024; Weidenbaum et al., 2025). Routine collection of sexual orientation and gender identity data can further support service planning, monitoring, and the identification of inequities in access and outcomes.

Finally, meaningful involvement of SGM communities in policy development, health care governance, and programme evaluation is critical to ensure that reforms are grounded in lived experience and responsive to community needs. Together, these measures can contribute to more affirming care environments, rebuild trust, and reduce health inequities over time.

### Strengths and Limitations

This study contributes novel evidence by integrating quantitative and qualitative data to examine discrimination in Spanish primary care, a context where disparities remain underexplored. The comparative design offers valuable insights into inequities within a universal health care system. Nevertheless, several limitations should be noted.

First, the cross-sectional design precludes causal inference. Second, the modest sample size, particularly within the SGM subgroup, may have limited statistical power in multivariate analyses, potentially reducing the ability to detect independent effects when multiple discrimination indicators were included. Therefore, non-significant regression findings should be interpreted with caution. Third, the qualitative component, although valuable for contextualizing quantitative results, was based on a limited number of responses and cannot capture the full diversity of experiences. Fourth, the use of convenience sampling introduces the potential for selection bias and limits the generalizability of the findings. This is particularly important given that the study examines healthcare discrimination, as individuals who participate may differ from broader population in their experiences of, or access to, healthcare. Finally, the sample was predominantly White and university-educated, which limits the generalizability of the findings. The study was also unable to examine intersectional dimensions such as race, socioeconomic status, or disability, due to limited representation of these groups. Future research should adopt explicitly intersectional and longitudinal designs to better capture how multiple forms of marginalisation shape health care discrimination and its cumulative effects over time.

### Conclusion

This study provides evidence that sexual and gender minority young adults in Spain experience disproportionate discrimination in primary care, despite universal health coverage and progressive legal protections. Perceived discrimination was associated with higher psychological distress, lower trust in providers, and delays in care, while qualitative accounts illustrated how bias is enacted in everyday encounters through practices such as misgendering, paternalism, and moral judgement. These findings underscore the limitations of policy frameworks in the absence of cultural competence, inclusive clinical practices, and institutional accountability. By addressing both provider-level and structural barriers, health systems may move towards delivering affirming and equitable care that fosters trust and supports the health of SGM populations.

## Data Availability

Data and materials are available upon request. Restrictions apply to the availability of these data due to concerns about participant privacy and data sensitivity.
